# Association of food insecurity with metabolic dysfunction-associated steatotic liver disease by sex and race/ethnicity

**DOI:** 10.1007/s00394-026-04021-8

**Published:** 2026-06-19

**Authors:** Meng-Hua Tao, Chun-Hui Lin, Jialiang Liu, Weiwen Chai, Sheri Trudeau, Humberto C. Gonzalez, Mei Lu, Stuart C. Gordon

**Affiliations:** 1 Department of Public Health Sciences, Henry Ford Health, Detroit, MI 48202 USA; 2 Department of Epidemiology and Biostatistics, Michigan State University, East Lansing, MI 48824 USA; 3 Quantitative Biomedical Research Center, University of Texas Southwestern Medical Center, Dallas, TX USA; 4 Department of Nutrition and Health Sciences, University of Nebraska-Lincoln, Lincoln, NE USA; 5 Department of Gastroenterology and Hepatology, Henry Ford Health, Detroit, MI USA; 6 Michigan State University School of Medicine, East Lansing, MI USA

**Keywords:** Metabolic-associated steatotic liver disease (MASLD), Food insecurity, Sex, Race/ethnicity

## Abstract

**Purpose:**

We examined whether the association between food insecurity and metabolic dysfunction-associated steatotic liver disease (MASLD) differs across different racial/ethnic groups and sex using data from the 2017–March 2020 National Health and Nutrition Examination Survey (NHANES).

**Methods:**

A total of 5076 participants aged ≥ 20 years who completed transient elastography examination for evaluation of MASLD were included. Logistic regression, odds ratios (ORs) and 95% confidence intervals (CIs) were used to estimate the association of food insecurity with MASLD.

**Results:**

Adult food insecurity was associated with increased odds of MASLD (low/very low vs. full security: OR = 1.41, 95% CI 1.12–1.77). Although there was no significant interaction between food insecurity and race/ethnicity (*p* interaction = 0.23), the positive association between food insecurity and MASLD was limited among non-Hispanic Whites. Food insecurity was associated with higher odds of MASLD in women but not men, with an interaction between food insecurity and sex (*p* interaction = 0.049). Furthermore, mediation analyses suggested that added sugar intake and intake of whole fruits and non-potato vegetables partially mediated the association of food insecurity with MASLD. The partial mediating effects of these foods were observed only among non-Hispanic Whites but not observed in other racial/ethnic groups or across sex.

**Conclusion:**

Our results suggest that the positive association between food insecurity and MASLD may be dependent on race/ethnicity and sex. Increased consumption of whole fruits vegetables intake and reduced intake of added sugar may partially reduce the impact of food insecurity on MASLD development.

**Supplementary Information:**

The online version contains supplementary material available at 10.1007/s00394-026-04021-8.

## Introduction

Metabolic-associated steatotic liver disease (MASLD) is the most common chronic liver disease, affecting over 30% of adults worldwide [1, 2]. MASLD defines a spectrum of hepatic steatosis with the presence of at least one of five known cardiometabolic risk factors, from hepatic steatosis to steatohepatitis, which can progress to advanced liver fibrosis and cirrhosis, liver failure, or even hepatocellular carcinoma [2]. In the US, disparities in prevalence of MASLD have become an important public health burden, with higher prevalence in males and Hispanics [3–5]. Several risk factors have been associated with the racial/ethnic variations in MASLD [6]; MASLD has also been recognized as a sex-dimorphic disease, which likely involves differences in sex hormones, immune responses, and metabolic processes between males and females [7]. However, detailed research on the association between social determinants of health (SDoH) and the risk of MASLD remains limited.

Food insecurity, an important SDoH, is defined as a condition of limited food availability for a healthy and active life [8], affecting 12.8% Americans in 2022 [8]. A few recent studies reported that adults with household food insecurity are more likely to have MASLD and advanced fibrosis compared to food-secure adults [9–12], and food insecurity is associated with increased all-cause mortality in adults with MASLD and advance fibrosis [13]. However, it remains unclear the role of sex and race/ethnicity as modifiers of the association between food insecurity and MASLD in a heterogeneous population of adults. Diet plays an important role in the development of MASLD [6]. Previous findings have shown that adults with food insecurity have lower intakes of fruits, vegetables and whole grains, and higher intakes of sugar-sweetened beverages [14, 15]. Because of the importance of dietary factors in MASLD development [6], food insecurity may increase the risk of MASLD through its influence on intakes fruits, vegetables and added sugar. Given the disease burden and disparities in MASLD, it is critical to disentangle the pathway from food insecurity to MASLD and consider the effective strategies to reduce the disease burden among adults in the US general population.

The aim of the present work was to evaluate whether the association between food insecurity and MASLD differs by sex and race/ethnicity, as well as apply mediation analysis to explore the contribution of fruits and vegetable, and added sugar intake to this association, using data from a representative sample of adults from the National Health and Nutrition Examination Surveys (NHANES) study.

## Materials and methods

### Study population

This study used data from continuous NHANES surveys conducted between 2017 and March 2020 pre-pandemic together with liver transient elastography examinations. The NHANES is designed to evaluate the health and nutrition status in a nationally representative sample of the noninstitutionalized U.S. population. A detailed description of the study deign has been published elsewhere [16]. The survey is maintained and administered by the National Center for Health Statistics (NCHS) of the Centers for Disease Control and Prevention (CDC) [16]. For NHANES 2017–March 2020, 15,560 participants (0–80 years) completed the interview with a 51.0% response rate [16]. All participants provided written informed consent and the NCHS Research Ethics Review Board approved the survey protocol (Protocol #2011-17, 2018-01). For this analysis, among 8004 adults aged > 19 who examined laboratory tests at a mobile examination center, we excluded 488 individuals for whom liver transient elastography data were ineligible or incomplete, as well as 505 individuals for whom laboratory tests for hepatitis B (HBV) or C virus (HCV) infection were ineligible. Participants with significant alcohol consumption, HBV or HCV infection, or autoimmune hepatic diseases, and/or pregnancy or breastfeeding were excluded. Participants for whom food security data were not available and had unreliable dietary recall data verified by trained study staff, or had unavailable data on alcohol intake were also excluded. We further excluded participants who had missing data on body mass index (BMI), physical activity, or demographic variables (age, sex, race/ethnicity, education, poverty income ratio). Finally, a total of 5076 participants were included in current analyses, with detailed selection process shown in Supplementary Fig. 1.

### Assessment of food insecurity

Food security was measured by the 10-item U.S. Adult Food Security Survey Module (FSSM), which were asked for households without children. All these FFSM items are listed in Supplementary Fig. 2. A 4-level adult food security status was calculated and assigned based on the number of affirmative responses to the 10 items of the FSSM using validated scoring as: (1) full food security (no affirmative responses); (2) marginal food security (1–2 affirmative responses); (3) low food security (3–5 affirmative responses for adult only and 3–7 affirmative responses for household); and (4) very low food security (6–10 affirmative responses for adult only) [17]. This study analyzed the data using both the categorical food insecurity variable with three categories (full, marginal, and low/very low food security) and the binary food insecurity variable (full/marginal, low/very low food security). We defined food insecurity as low and very low food security.

### Defining steatotic liver disease (SLD) and its subtypes

In the 2017–March 2020 cycles of NHANES, liver ultrasound transient elastography measurements were performed by FibroScan® (Echosens, Cambridge, MA) in the NHANES Mobile Examination Center (MEC). The device includes a novel physical parameter, controlled attenuation parameter (CAP™; Echosens, Cambridge, MA), which measures the ultrasound attenuation related to the presence of steatotic liver disease (SLD). Only participants with complete exams (i.e., fasting time of at least 3 h, ≥ 10 complete stiffness measures, and a liver stiffness interquartile range/median < 30%) were included in the current analysis. The procedure for this examination has been detailed in the NHANES Liver Ultrasound Transient Elastography Procedures Manual [18]. We used 263 dB/m (> S1) as the CAP cut-off value to define participants with SLD [2]. In addition, SLD was defined as CAP cut-off value of 285 for sensitivity analysis [2]. We used 8.6 kPa as liver stiffness cut-off values to determine clinically significant fibrosis [19].

In the presence of SLD, participants were to have MASLD if they met at least one of the following cardiometabolic risk criteria: (1) BMI > 25 (weight (kg)/height (m)^2^) or waist circumference > 94 cm (male [M]) or > 80 cm (Female [F]); (2) HbA1c > 5.7% or history of diabetes; 3) blood pressure ≥ 130/85 mmHg or history of high blood pressure; (4) plasma HDL-cholesterol levels ≤ 1.0 mmol/L (M) or ≤ 1.3 mmol/L (F); and (5) triglyceride level ≥ 150 mg/dL [2].

### Assessment of dietary intake

Foods and alcohol consumption data was collected via 24 h dietary recall interviews [20]. The first interview was administered in person by trained interviewers in NHANES MEC and the second was completed by trained interviewers via telephone 3–10 days after the MEC interview [20]. Food groups data was stored in the Food Patterns Equivalents Database (FPED) developed by the Food Surveys Research Group of the US Department of Agriculture (USDA) in NHANES [21]. Only the in-person recall data was used in the present analyses. Consumption of whole fruits, non-potato vegetables, and added sugar (WF/V/S) was calculated by using cycle-specific version of the USDA food composition database [21].

### Statistical analyses

Characteristics of participants were compared between those with and without MASLD using Rao-Scott chi-square test for categorical variables and two-sample t-test for continuous variables. The age-adjusted prevalence of MASLD and food insecurity were estimated, stratified by age groups using the 2000 US Census as the standard population.

Logistic regression was applied to estimate odds ratios (OR) and 95% confidence intervals (95% CI) for the associations between adult food security level and MASLD. We considered a number of factors as potential confounding factors including: age; biological sex (male, female); race/ethnicity (non-Hispanic White (NHW), non-Hispanic Black (NHB), Hispanics, and Others); educational level (high school and less, some college, college and high); poverty income ratio (PIR: < 1.30, 1.30– < 1.85, and ≥ 1.85); marital status (married/living with partner, single/divorced/widow); cigarette smoking status (never, former, current); physical activity level (physical active, moderate physical active, inactive); and history of heart disease (i.e., coronary heart disease, stroke, heart attack, chronic obstructive pulmonary disease (COPD), or heart failure) (yes, no). The analyses considered possible modification effects of sex and race/ethnicity in the logistic regression model by evaluation of a multiplicative term (e.g., adult food insecurity × sex or adult food insecurity × race/ethnicity). In addition, we repeated the above analyses, stratified by participants’ alcohol intake levels: low alcohol consumption (F: < 20 g/day; M < 30 g/day) and moderate alcohol consumption (F: 20–50 g/day; M: 30–60 g/day) [2]. We also repeated the above analyses, stratified by participants’ liver stiffness: with (> 8.6 kPa) and without (≤ 8.6 kPa) clinically significant fibrosis.

To study a possible mediation effect, multi-variable adjusted logistic structural equation modeling with the bootstrapping method was conducted and the models included a direct effect (DE) path from the exposure variable (food insecurity) to the outcome variable (MASLD), as well as an indirect effect (IE) path from the exposure variable to the mediators (e.g., total intake of whole fruits and non-potato vegetables, intake of added sugar), and from the mediators to the outcome [22]. Mediation proportion, ratio of IE to TE, showed the degree to which the total effect is mediated through the mediator. The direct (OR^DE^), indirect (OR^IE^) and total (OR) effects were deemed significant if the corresponding 95% CIs of ORs did not contain 1.

All analyses used sampling weights and clusters to incorporate the complex, multistage clustered survey sampling design of the NHANES [16]. All statistical tests were 2-sided with a significance level of 0.05. The ‘Survey’ procedure in SAS version 9.4 (SAS Institute Inc, Cary, NC) was used to conduct the descriptive analyses and logistic regression analyses, and the mediation analyses were conducted using the ‘svy’ procedure in Stata release 18.0 (Stata Corporation, College Station, TX).

## Results

Of 5076 participants included in the study, 2627 participants met the definition of MASLD with the CAP cut-off value of 263 dB/m. The age-adjusted prevalence of adult food insecurity (low/very low food security) was 15.4%, with higher prevalence in participants with MASLD than those without (17.9% vs. 13.4%). Table 1 summarizes characteristics by MASLD status. Overall, compared to participants without MASLD, those with MASLD were older, more likely to be males, Hispanics, single/divorced/widow, former smokers, physical inactive, and to have lower education levels, a history of heart diseases, less consumption of whole fruits and non-potato vegetables (WF/V), and higher intake of added sugar.

**Table 1:** Participant characteristics by MASLD status, NHANES 2017–March 2020

	Total sample (*n* = 5076)	MASLD status^1^		*p*-value
		Yes (*n* = 2627)	No (*n* = 2449)	
Age (years)^ǂ^	47.7 (0.76)	50.7 (0.80)	44.7 (0.83)	< 0.001
Sex, *n* (%)*				< 0.001
Male	2444 (48.4)	1366 (53.2)	1078 (43.7)	
Female	2632 (51.6)	1261 (46.8)	1371 (56.3)	
Race/ethnicity, *n* (%)*				< 0.001
Non-Hispanic White	2010 (67.4)	1069 (66.7)	941 (68.2)	
Non-Hispanic Black	1222 (9.6)	542 (8.2)	680 (11.1)	
Hispanic	1126 (14.8)	667 (16.8)	459 (12.8)	
Others^§^	718 (8.1)	349 (8.3)	369 (8.0)	
Educational level, *n* (%)*				< 0.001
High school or less	1975 (34.9)	1086 (38.2)	889 (31.7)	
Some college	1769 (31.5)	928 (33.3)	841 (29.6)	
College and above	1332 (33.6)	613 (28.5)	719 (38.7)	
Poverty income ratio, *n* (%)*				0.70
< 1.30	1307 (16.5)	665 (16.5)	642 (16.5)	
1.30– ≤ 1.85	690 (9.5)	376 (9.8)	314 (9.1)	
> 1.85	3079 (74.0)	1586 (73.7)	1493 (74.4)	
Marital status, *n* (%)				< 0.001
Single/separate/widow	2091 (37.0)	984 (31.7)	1107 (42.2)	
Married/being with partner	2983 (63.0)	1642 (68.3)	1341 (57.8)	
Smoking status, *n* (%)*				< 0.001
Never	2994 (59.0)	1487 (56.5)	1507 (61.4)	
Former	1114 (23.1)	684 (27.2)	430 (19.1)	
Current	968 (17.9)	456 (16.3)	512 (19.5)	
Physical activity, *n* (%)*				< 0.001
Physical inactive	2562 (43.0)	1475 (50.3)	1087 (35.7)	
Moderate physical active	1600 (34.5)	806 (34.0)	794 (35.1)	
Physical active	914 (22.5)	346 (15.7)	568 (29.3)	
History of heart disease^†^, *n* (%)*				< 0.001
No	4247 (86.1)	2135 (82.9)	2112 (89.2)	
Yes	829 (13.9)	492 (17.1)	337 (10.8)	
Adult food insecurity, *n* (%)*				0.135
Very low food security	488 (6.7)	254 (6.9)	234 (6.4)	
Low food security	602 (8.4)	345 (9.5)	257 (7.2)	
Marginal food security	698 (10.5)	357 (10.7)	341 (10.3)	
Full food security	3288 (74.5)	1671 (72.8)	1617 (76.1)	
Daily intake of foods				
Added sugars (g)	70.6 (2.0)	76.1 (2.3)	65.2 (2.2)	< 0.001
Whole fruits and non-potato vegetables^‡^ (cups)	1.90 (0.07)	1.80 (0.06)	2.00 (0.10)	0.025

^1^We defined MASLD with controlled attenuation parameter (CAP) ≥ 263 dB/m

*Unweighted frequency (weighted percentage)

^ǂ^Weighted mean (standard error)

^§^Others: non-Hispanic Asians, and other non-Hispanic races including multiple racial persons

^†^Heart diseases include coronary heart disease, heart attack, stroke, heart failure, or chronic obstructive pulmonary disease (COPD)

^‡^Non-potato vegetables including dark green vegetables, total red and orange vegetables, other starchy vegetables, other vegetables, and legumes

Association between adult food security and risk of MASLD are presented in Table 2. Compared to those with full/marginal food security, participants with food insecurity had higher odds of MASLD (OR = 1.32, 95% CI 1.05–1.66) after adjustment for age, sex, race/ethnicity, and other confounding factors. When classifying food security into three categories (full, marginal, low/very low food security), lower level of food security was also associated with higher odds of MASLD, the corresponding ORs and 95% CIs for marginal food security and food insecurity vs. full food security were 1.29 (1.03–1.60) and 1.41 (1.12–1.77), respectively. Sensitivity analyses used CAP cut-off value of 285 dB/m to define MASLD, and similar but slightly attenuated associations were observed (Supplementary Table 1), partly due to a smaller number of participants identified as prevalent MASLD cases.

**Table 2:** Overall and sex-specific associations between adult food security status and MASLD in NHANES 2017-March 2020*

Population		MASLD		Model 1	Model 2
		Yes	No		
Overall	Adult food security				
	Full	1670	1616	1.00^a^	1.00^c^
	Marginal	357	341	1.30 (1.01–1.67)	1.29 (1.03–1.60)
	Low/very low	599	491	1.46 (1.18–1.81)	1.41 (1.12–1.77)
	Adult food security				
	Full/marginal	2027	1957	1.00^a^	1.00^c^
	Low/very low	599	491	1.39 (1.18–1.65)	1.35 (1.15–1.59)
Female	Adult food security				
	Full	754	916	1.00^b^	1.00^c^
	Marginal	190	181	1.59 (1.22–2.08)	1.45 (1.15–1.83)
	Low/very low	316	273	1.58 (1.23–2.04)	1.45 (1.08–1.95)
	Adult food security				
	Full/marginal	944	1097	1.00^b^	1.00^c^
	Low/very low	316	273	1.46 (1.13–1.89)	1.32 (0.97–1.78)
Male	Adult food security				
	Full	916	700	1.00^b^	1.00^c^
	Marginal	167	160	1.02 (0.70–1.49)	1.03 (0.71–1.48)
	Low/very low	283	218	1.34 (0.97–1.85)	1.33 (0.91–1.95)
	Adult food security				
	Full/marginal	1083	860	1.00^b^	1.00^c^
	Low/very low	283	218	1.33 (0.97–1.83)	1.32 (0.91–1.91)

*We defined MASLD with controlled attenuation parameter (CAP) ≥ 263 dB/m

^a^Adjusted for age, sex, race/ethnicity

^b^Adjusted for age, race/ethnicity

^c^Additionally adjusted for educational level, poverty income ratio, marital status, smoking status, physical activity, history of heart disease (i.e., coronary heart disease, heart attack, stroke, heart failure, or COPD)

The association between adult food security and MASLD varied across sex and race/ethnicity. Potential interaction effects were identified between food insecurity and sex for MASLD (*p* interaction = 0.049) (Table 2). Food insecurity was significantly associated with higher odds of MASLD in females (compared to full food security: OR = 1.45, 95% CI 1.08–1.95) but not in males. When compared to females with full/marginal food security, the association between food insecurity and MASLD was attenuated and became marginally significant. Positive associations between food insecurity and odds of MASLD were observed in NHWs (compared to full food security: OR = 1.59, 95% CI 1.15–2.21), but not statistically significant in other racial/ethnic groups due to limited sample size. When compared to full/marginal food security, the positive association between food insecurity and MASLD persisted in NHWs. However, there was no interaction between food insecurity and race/ethnicity for MASLD (*p* interaction = 0.23) (Table 3).

**Table 3:** Race-specific associations between adult food security status and MASLD in NHANES 2017–March 2020*

	NH White		NH Black		Hispanic		Others^§^	
	Cases/total	OR (95% CI)	Cases/total	OR (95% CI)	Cases/total	OR (95% CI)	Cases/total	OR (95% CI)
Adult food security								
Full	777/1489	1.00	308/691	1.00	339/583	1.00	247/525	1.00
Marginal	106/192	1.62 (1.20–2.18)	97/227	0.96 (0.63–1.46)	120/201	1.11 (0.68–1.80)	34/78	0.86 (0.45–1.65)
Low/very low	186/329	1.59 (1.15–2.21)	137/304	1.03 (0.71–1.51)	208/342	1.16 (0.70–1.95)	68/115	1.64 (0.87–3.08)
Adult food security								
Full/marginal	883/1681	1.00	405/918	1.00	459/784	1.00	281/603	1.00
Low/very low	186/329	1.44 (1.03–2.01)	137/304	1.04 (0.73–1.49)	208/342	1.12 (0.73–1.73)	68/115	1.71 (0.92–3.17)

We defined MASLD with controlled attenuation parameter (CAP) ≥ 263 dB/m

*Adjusted for age, sex, educational level, poverty income ratio, marital status, smoking status, physical activity, history of heart disease (i.e., coronary heart disease, heart attack, stroke, heart failure, or COPD)

^§^Others: non-Hispanic Asians, other non-Hispanic races including non-Hispanic multiple racial persons

In analyses stratified by presence or absence of clinically significant fibrosis (Supplementary Table 2), a positive association between food insecurity and MASLD was observed among participants without clinically significant fibrosis (≤ 8.6 kPa). Among individuals with clinically significant fibrosis (> 8.6 kPa), there was a trend towards a positive association between food insecurity and MASLD, but results did not reach statistical significance due to small sample size. In further analyses stratified by alcohol consumption, a similar pattern was observed for participants with low alcohol consumers but not among moderate alcohol consumers.

We estimated that intake of WF/V/S mediated 9.1% of the association between food insecurity (low/very vs. full food security) and MASLD in the overall population with statistically significant indirect association (OR^IE^ = 1.032, 95% CI 1.007–1.058) (Table 4). We observed that intake of WF/V/S mediated 11.1% of the food insecurity-MASLD association in NHWs, and the indirect association was statistically significant (OR^IE^ = 1.054, 95% CI 1.007–1.102, *p* = 0.023). A non-significant indirect association mediated through WF/V/S intake was observed in females, with OR^IE^ of 1.015 (95% CI 0.990–1.040) and mediated proportion of 3.8%. In addition, we observed that intake of WF/V/S mediated 11.3% and 16.1% of the association between marginal food security and MASLD in the overall population and NHWs, respectively. We did no find evidence for mediation through intake of WF/V/S for food security status (i.e., marginal vs. full security; low/very low vs. full security) and MASLD in males or other racial/ethnic groups.

**Table 4:** Association between food security status and MASLD mediated through intakes of whole fruits, non-potato vegetables and added sugars*

Population	Indirect effect OR^IE^ (95% CI)	Direct effect OR^DE^ (95% CI)	Total effect OR^TE^ (95% CI)
Overall population^a^			
Full	Reference		
Marginal	1.029 (1.004–.054)	1.25 (1.001–1.560)	1.285 (1.038–1.592)
Low/very low	1.032 (1.007–1.058)	1.368 (1.091–1.715)	1.412 (1.124–1.774)
Females^b^			
Full	Reference		
Marginal	1.017 (0.993–1.041)	1.433 (1.145–1.793)	1.457 (1.165–1.823)
Low/very low	1.015 (0.990–1.040)	1.441 (1.077–1.927)	1.462 (1.084–1.971)
Males^b^			
Full	Reference		
Marginal	1.035 (0.984–1.088)	0.99 (0.667–1.467)	1.024 (0.698–1.503)
Low/very low	1.049 (0.990–1.110)	1.266 (0.871–1.838)	1.327 (0.909–1.938)
NH White^c^			
Full	Reference		
Marginal	1.081 (1.01, 1.156)	1.5 (1.064–2.116)	1.621 (1.172–2.244)
Low/very low	1.054 (1.007, 1.102)	1.519 (1.074–2.147)	1.601 (1.126–2.275)
NH Black^c^			
Full	Reference		
Marginal	1.007 (0.977–1.037)	0.952 (0.601–1.511)	0.959 (0.609–1.510)
Low/very low	1.003 (0.847–1.023)	1.027 (0.686–1.537)	1.030 (0.688–1.542)
Hispanic^c^			
Full	Reference		
Marginal	1.021 (0.974–1.069)	1.087 (0.655–1.803)	1.11 (0.683–1.801)
Low/very low	1.029 (0.801–1.084)	1.133 (0.672–1.910)	1.166 (0.690–1.972)
Others^c§^			
Full	Reference		
Marginal	1.0 (0.989–1.010)	0.864 (0.399–1.870)	0.863 (0.399–1.867)
Low/very low	1.003 (0.477–1.084)	1.634 (0.792–3.368)	1.639 (0.806–3.334)

We defined MASLD with controlled attenuation parameter (CAP) ≥ 263 dB/m

*Adjusted for age, educational level, poverty income ratio, marital status, smoking status, physical activity, history of heart disease (i.e., coronary heart disease, heart attack, stroke, heart failure, or COPD)

^a^Additionally adjusted sex and race/ethnicity

^b^Additionally adjusted race/ethnicity

^c^Additionally adjusted sex

^§^Others: non-Hispanic Asians, other non-Hispanic races including non-Hispanic multiple racial persons

## Discussion

Using recent NHANES data, we found that food insecurity was associated with increased odds of MASLD among US adults. Our analysis also showed that this positive association was dependent on sex and race/ethnicity, with stronger associations in women than in men, and in NHWs than in other racial/ethnic groups. To our knowledge, this study was the first to investigate sex and race/ethnicity-specific associations between food insecurity and the updated definition of MASLD in a representative sample of the US population.

Consistent with previous studies [9–12], our results supported independent positive associations between food insecurity and MASLD. However, Kardashian et al. [23] recently observed association between food insecurity and lower odds of NAFLD among people with HIV (PWH). This inconsistency may be due to significant demographic differences between the two study populations considering that PWH population includes over 70% males and 25% NHW. Variations may be explained, partially, by the differences in definitions for MASLD and NAFLD. The study showed that among PWH with type 2 diabetes, food insecurity was associated with increased odds of advanced fibrosis [23].

The positive association of adult food insecurity with MASLD was limited among NHWs but not statistically significant in other racial/ethnic groups in our study. Food insecurity can impact diet quality [14, 15], while this relationship differs by race/ethnicity [24, 25], possible reflecting different patterns of foods selections [24, 26]. Studies found significantly lower diet quality in food-insecure NHWs than food-secure NHWs; however, diet quality did not differ significantly between food-secure NHBs and Hispanics and their food-insecure counterparts [24]. It is possible that food secure NHBs and Hispanics still have limited access to nutritious foods, which may at least partially interpret the observed non-significant associations with MASLD among NHBs and Hispanics in our study. Our results suggest that reducing racial/ethnic disparities in MASLD may require efforts to improve access to healthful foods, and nutritional education regarding affordably nutritious food options [27, 28] especially in NHB and Hispanic neighborhoods. Although minorities were oversampled in the NHANES 2017–March 2020 cycle [16], we cannot rule out that we had limited power to detect weak associations in racial/ethnic groups. Further studies with large sample size of minorities are warranted to elucidate the association between food insecurity and MASLD in racial/ethnic groups, such as NHBs and Hispanics.

We also found that food insecurity was significantly associated with odds of MASLD in females but not in males, and there was a significant interaction between sex and food insecurity on MASLD. Studies have identified a number of sex variations in factors related to both food access and intake [29, 30] and risk of MASLD [3, 31]. Several gender-dimorphic risk factors of MASLD including sex hormone levels, obesity and dysmetabolic traits have been identified [7]. However, sex as a modifier of the association between food insecurity and MASLD has not been analyzed in epidemiological studies to date [9–12]. Additional studies of the interplay between sex, food insecurity, and MASLD are needed to better inform prevention and treatment for MASLD.

The relationship between dietary factors and MASLD pathophysiology is well known [32, 33]. Our mediation analyses suggested that the association of food insecurity with odds of MASLD in the overall population may be partially explained by its relationship with intakes of WF/V/S. Of note, we observed a stronger association of food insecurity with intakes of WF/V/S and MASLD among NHWs than among other races/ethnicities. In the US, differences in food consumption exist across racial/ethnic groups: i.e., NHBs have higher added sugar intake and lower whole fruits and vegetables intake than NHW and Hispanic individuals [34, 35]. Additionally, racial/ethnic differences in the association between food insecurity and consumption of different foods have been previously reported, for example, food-insecure NHW reported consuming more added sugar and fewer fruits, vegetables, or plant proteins than their counterparts. However, consumption of foods did not vary significantly across different food-secure status in NHBs or Hispanics [24, 26, 36]. Although we need to develop an effective strategy to mitigate racial/ethnic disparities in MASLD, our findings indicate that, even among NHW, the food insecurity may contribute to the increased risk of MASLD mediated through intakes of WF/V/S.

Our findings have significant clinical and public health implications. First, the higher age-adjusted prevalence of food insecurity in adults with MASLD than that of the general population without MASLD highlights that lack of access to food is an important public health issue in people with MASLD. Additionally, the observed association is consistent with prior studies, supporting that food insecurity may be an independent modifiable risk factor of MASLD. In clinical practice, clinicians should enhance to screen food insecurity for their MASLD patients. Given the mediation role of WF/V/S on the association of food insecurity with MASLD, patients who are identified as experiencing or at risk of food insecurity may benefit from precise case management and food assistance programs. At the community level, greater efforts are needed to develop targeted nutrition education programs or plans and promote access to affordably healthy food, particularly in vulnerable populations.

We acknowledge several limitations common to observational studies. The cross-sectional study design could not determine the temporal sequence of the observed associations. However, the positive associations between food insecurity and risk of MASLD are consistent with previous studies [9–13]. Another limitation is that there is no consensus on the best measures for diagnosis of MASLD; to address this, we used the previously validated cut-off points [2]. Although adult food insecurity was assessed with the well-validated US FSSM [17], there is always risk of recall bias from self-reported questionnaire. In addition, not every participant of NHANES 2017-March 2020 had data on liver transient elastography and food security; however, it is unlikely that unavailable data of food security would be related to examine liver transient elastography and thus would not differentially affect our results. Finally, our study can be limited by the self-reported dietary recall data for daily alcohol consumption and intake of foods which may have random and systematic errors with potential recall bias.

In conclusion, food insecurity is associated with increased odds of MASLD in adults, and total intake of WF/V/S partially mediated the association. The positive association between food insecurity and MASLD is stronger in NHW and females, compared to other racial/ethnic groups and males, respectively. Future prospective studies, with large racially/ethnically diverse samples and balanced proportions of men and women are warranted to confirm these findings.

## Supplementary Information

Below is the link to the electronic supplementary material.

Supplementary Material 1

## Data Availability

Data used in this study can be accessed through NHANES website, and all NHANES study materials are publicly available at: NHANES Questionnaires, Datasets, and Related Documentation.
