# Supplementary material for: Association of food insecurity with metabolic dysfunction-associated steatotic liver disease by sex and race/ethnicity
Source: Eur J Nutr. 2026 Jun 19;65(5):172. doi: 10.1007/s00394-026-04021-8 (PMC13282211; doi:10.1007/s00394-026-04021-8)
Supplement: Supplementary file 1 — Supplementary Material 1 [file 394_2026_4021_MOESM1_ESM.docx]

**Supplemental Material**

15,560 Participants

- Total number of participants completed interview
- No exclusions
- 7,556 excluded for age < 19 years or without laboratory test

8,004 Participants

- 488 excluded for ineligible or incomplete data on liver transient elastography,

505 excluded for missing data on hepatitis B (HBV) or C virus (HCV) infection,

499 excluded for having significant alcohol consumption, HBV or HCV infection,

or autoimmune hepatic diseases, and/or pregnancy or breastfeeding

6,512 Participants

- 614 excluded for missing data on food security, and unreliable dietary recall,

408 excluded for missing data on alcohol intake

5,490 Participants

- 414 excluded for missing data on BMI, physical activity, or demographic variables

5,076 Participants

**Supplementary Figure 1.** Study Participants Flow Chart

**Supplementary Figure 2.** 10-item Adult Food Security Survey Module (FSSM)

*Affirmative responses are “often true” or “sometimes true” to one or more of HH2- HH4, then continue to Adults Stage Questions.*

HH2. I worried whether my food would run out before I got money to buy more. Was that often, sometimes, or never true for (you/your household) in the last 12 months?

HH3. The food that I bought just didn’t last, and I didn’t have enough money to get more. Was that often, sometimes, or never true for (you/your household) in the last 12 months?

HH4. I couldn’t afford to eat balanced meals. Was that often, sometimes, or never true for (you/your household) in the last 12 months?

*Adult Stage Questions:*

AD1. In the last 12 months, did you ever cut the size of your meals or skip meals because there wasn’t enough money for food?

AD1a. How often did this happen – almost every month, some months but not every month, or in only 1 or 2 months?

AD2. In the last 12 months, did you ever eat less than you felt you should because there wasn’t enough money for food?

AD3. In the last 12 months, were you ever hungry but didn’t eat because there wasn’t enough money for food?

AD4. In the last 12 months, did you lose weight because there wasn’t enough money for food?

AD5. In the last 12 months, did you ever not eat for a whole day because there wasn’t enough money for food?

AD5a. How often did this happen – almost every month, some months but not every month, or in only 1 or 2 months?

**Supplementary Table 1.** Overall associations between adult food insecurity status and MASLD in NHANES 2017-March 2020 ^*^

|  | MASLD | |  |  |
| --- | --- | --- | --- | --- |
|  | Yes | No | Model 1 | Model 2 |
| Adult food security |  |  |  |  |
| Full | 1256 | 2032 | 1.00 | 1.00 |
| Marginal | 275 | 423 | 1.38 (1.04-1.83) | 1.33 (1.02-1.73) |
| Low/ very low | 454 | 636 | 1.31 (1.06-1.61) | 1.21 (0.97-1.52) |
| Adult food security |  |  |  |  |
| Full/marginal | 1531 | 2455 | 1.00 | 1.00 |
| Low/very low | 454 | 636 | 1.24 (1.00-1.54) | 1.12 (0.88-1.43) |

^*^ We defined MASLD with controlled attenuation parameter (CAP) ≥ 285 dB/m.

Model 1 adjusted for age, sex, race/ethnicity; Model 2 additionally adjusted for educational level, poverty income ratio, marital status, smoking status, physical activity, history of heart disease (i.e., coronary heart disease, heart attack, stroke, heart failure, or COPD).

**Supplementary Table 2.** Associations of adult food insecurity with MASLD, by alcohol consumption and clinically significant fibrosis status

|  | Low alcohol intake  (F: <20 g/day; M: < 30g/day) | | Moderate alcohol intake  (F: 20-50 g/day; M: 30-60 g/day) | |
| --- | --- | --- | --- | --- |
|  | MASLD: Yes/No | OR (95% CI) ^a^ | MASLD: Yes/No | OR (95% CI) ^a^ |
| Adult food security |  |  |  |  |
| Full | 1590/1551 | 1.00 | 81/66 | 1.00 |
| Marginal | 341/323 | 1.32 (1.09-1.60) | 16/18 | 0.63 (0.15-2.61) |
| Low/very low | 566/464 | 1.37 (1.07-1.74) | 33/27 | 1.48 (0.51-4.29) |
| Adult food security |  |  |  |  |
| Full /marginal | 1931/1874 | 1.00 | 97/84 | 1.00 |
| Low/very low | 566/464 | 1.27 (0.99-1.63) | 33/27 | 1.68 (0.66-4.28) |
|  | Without clinically significant fibrosis  (≤ 8.6 kPa) | | With clinically significant fibrosis  (> 8.6 kPa) | |
|  | MASLD: Yes/No | OR (95% CI) ^a^ | MASLD: Yes/No | OR (95% CI) ^a^ |
| Adult food security |  |  |  |  |
| Full | 1429/1570 | 1.00 | 242/47 | 1.00 |
| Marginal | 299/328 | 1.26 (1.01-1.58) | 58/13 | 0.78 (0.20-3.06) |
| Low/very low | 511/481 | 1.41 (1.11-1.78) | 88/10 | 2.26 (0.60-8.54) |
| Adult food security |  |  |  |  |
| Full /marginal | 1728/1898 | 1.00 | 300/60 | 1.00 |
| Low/very low | 511/481 | 1.32 (1.06-1.66) | 88/10 | 2.41 (0.72-8.03) |

We defined MASLD with controlled attenuation parameter (CAP) ≥ 263 dB/m.

^a^ Adjusted for age, sex, race/ethnicity, educational level, poverty income ratio, marital status, smoking status, physical activity, and heart disease history disease (i.e., coronary heart disease, heart attack, stroke, heart failure, or COPD).
